# BCECNN: an explainable deep ensemble architecture for accurate diagnosis of breast cancer

**DOI:** 10.1186/s12911-025-03186-2

**Published:** 2025-10-13

**Authors:** Uçman Ergün, Tuğçe Çoban, İsmail Kayadibi

**Affiliations:** 1 Department of Biomedical Engineering, Faculty of Engineering, Afyon Kocatepe University, Afyonkarahisar, Turkey; 2 Department of Management Information Systems, Faculty of Economics and Administrative Sciences, Afyon Kocatepe University, Afyonkarahisar, Turkey

**Keywords:** Breast cancer, Deep learning, Convolutional neural networks, Ensemble learning, Explainable artificial intelligence

## Abstract

**Background:**

Breast cancer remains one of the leading causes of cancer-related deaths globally, affecting both women and men. This study aims to develop a novel deep learning (DL)-based architecture, the Breast Cancer Ensemble Convolutional Neural Network (BCECNN), to enhance the diagnostic accuracy and interpretability of breast cancer detection systems.

**Methods:**

The BCECNN architecture incorporates two ensemble learning (EL) structures: Triple Ensemble CNN (TECNN) and Quintuple Ensemble CNN (QECNN). These ensemble models integrate the predictions of multiple CNN architectures—AlexNet, VGG16, ResNet-18, EfficientNetB0, and XceptionNet—using a majority voting mechanism. These models were trained using transfer learning (TL) and evaluated on five distinct sub-datasets generated from the Artificial Intelligence Smart Solution Laboratory (AISSLab) dataset, which consists of 266 mammography images labeled and validated by radiologists. To improve transparency and interpretability, Explainable Artificial Intelligence (XAI) techniques, including Gradient-weighted Class Activation Mapping (Grad-CAM) and Local Interpretable Model-Agnostic Explanations (LIME), were applied. Additionally, explainability was assessed through clinical evaluation by an experienced radiologist.

**Results:**

Experimental results demonstrated that the TECNN model—comprising AlexNet, VGG16, and EfficientNetB0—achieved the highest accuracy of 98.75% on the AISSLab-v2 dataset. The integration of XAI methods substantially enhanced the interpretability of the model, enabling clinicians to better understand and validate the model’s decision-making process. Clinical evaluation confirmed that the XAI outputs aligned well with expert assessments, underscoring the practical utility of the model in a diagnostic setting.

**Conclusion:**

The BCECNN model presents a promising solution for improving both the accuracy and interpretability of breast cancer diagnostic systems. Unlike many previous studies that rely on single architectures or large datasets, BCECNN leverages the strengths of an ensemble of CNN models and performs robustly even with limited data. It integrates advanced XAI techniques—such as Grad-CAM and LIME—to provide visual justifications for model decisions, enhancing clinical interpretability. Moreover, the model was validated using AISSLab dataset, designed to reflect real-world diagnostic challenges. This combination of EL, interpretability, and robust performance on small yet clinically relevant data positions BCECNN as a novel and reliable decision support tool for AI-assisted breast cancer diagnostics.

## Introduction

Breast cancer is the most frequently diagnosed cancer among women worldwide and remains one of the leading causes of cancer-related deaths. According to Global Cancer Observatory (GLOBOCAN)—an initiative of the International Agency for Research on Cancer (IARC), which is the specialized cancer agency of the World Health Organization (WHO)—an estimated 2.3 million new cases and 670,000 deaths due to breast cancer occurred globally. Breast cancer accounts for approximately one in four cancer diagnoses and one in six cancer deaths among women, underscoring its critical impact on global women’s health [1].

Despite the availability of various imaging modalities such as Magnetic Resonance Imaging (MRI) and ultrasound for breast cancer detection, mammography remains the most widely used method [2]. In mammography, the large volume of scans contributes to the inherent subjectivity of visual interpretation [3]. However, accurate diagnosis is critical for the timely and effective treatment of breast cancer [4]. To overcome these challenges in mammographic imaging, recent efforts have focused on developing computer-assisted decision support systems. In this context, Artificial Intelligence (AI), particularly deep learning (DL) techniques, has recently emerged as a powerful tool in medical image analysis [5].

AI systems are designed to mimic human intelligence in performing tasks, while DL methods, particularly Convolutional Neural Network (CNN)s, are capable of learning complex patterns in imaging data. CNNs have demonstrated remarkable success in image processing tasks and are now widely adopted across medical imaging modalities, including MRI, CT, and X-ray [6, 7]. Their ability to rapidly and accurately analyze large volumes of imaging data has made them invaluable for the early detection of anomalies such as cancer, thereby contributing to improved diagnostic efficiency and patient outcomes [8].

Among the DL methods used in breast cancer diagnosis, CNNs are frequently preferred because they achieve successful results with high accuracy rates. These methods are important for the early detection and accurate classification of cancer. However, due to the limited amount of data in the field of medical imaging, TL methods are often preferred. TL makes it possible to achieve high accuracy rates even in limited datasets by reusing pre-trained architectures [9]. High accuracy rates alone are not sufficient, so Explainable Artificial Intelligence (XAI) techniques are used to visualize AI decision-making mechanisms and make them more interpretable for experts [10]. These techniques such as gradient-weighted class activation mapping (Grad-CAM) [11] and local interpretable model-agnostic explanations (LIME) [12] are also considered powerful tools for increasing user confidence in AI systems by visualizing the internal decision-making processes of DL architectures [13].

In this study, a novel Breast Cancer Ensemble CNN (BCECNN) architecture is proposed, which combines TL-based CNN architectures with an Ensemble Learning (EL) strategy for breast cancer detection. The proposed methodology integrates multiple CNN models using a majority voting-based EL approach. As established in the literature, ensemble strategies effectively address the performance limitations of individual CNN models, yielding more balanced and generalizable outcomes [14]. While traditional single CNN-based models may achieve high accuracy on specific datasets, they often suffer from overfitting and may lack the ability to generalize across diverse patient populations [15, 16]. To mitigate these challenges, combining the outputs of multiple models can lead to more robust and reliable decision-making [16]. Furthermore, the integration of XAI techniques into DL architectures enhances transparency by elucidating the model’s decision-making process beyond conventional performance metrics [17]. In this context, visualization methods such as Grad-CAM, LIME, and LIME-Mask were employed within the BCECNN framework to demonstrate how the model interprets and predicts breast cancer from mammographic images using high-performing CNN architectures.

This study advances beyond existing DL approaches, which often achieve high accuracy in breast cancer diagnosis but lack explainability, by prioritizing model reliability, transparency, and clinical applicability. While many models in the literature report high accuracy rates, most do not provide interpretable insights into their decision-making processes, hindering the integration of AI-based diagnostic systems into clinical workflows. To address these limitations, the proposed BCECNN architecture is designed to deliver both high diagnostic accuracy and transparent decision-making, thereby laying a robust foundation for future clinical decision support systems. The main contributions of this study are summarized below.

1. Conducted t-SNE analysis on the Artificial Intelligence Smart Solution Laboratory (AISSLab) dataset to create five distinct versions (v1–v5), enabling diverse classification scenarios that have not yet been explored in the literature.
2. Trained pre-trained CNN architectures—AlexNet, VGGNet-16, ResNet18, EfficientNetB0, and XceptionNet—on the AISSLab dataset using TL, followed by a comparative evaluation based on performance metrics.
3. Proposed a novel EL-based architecture, BCECNN, for the accurate detection of breast cancer in mammograms images.
4. Generated XAI visualizations, including heatmaps of the CNN models used in the proposed architecture, utilizing techniques such as Grad-CAM, LIME, and LIME-Mask.
5. Compared the proposed architecture with recent state-of-the-art approaches reported in the literature.

The remainder of this paper is structured as follows. A comprehensive literature review on the subject is presented in Sect. 2. Then, in Sect. 3, an overview of the proposed method, the characteristics of the AISSLab dataset, the applied image preprocessing techniques, DL methods, the pre-trained CNN architectures used, Grad-CAM, LIME, and LIME-Mask techniques, and the proposed BCECNN architecture are described in detail. Section 4 presents the experiments conducted to evaluate the performance of the proposed method and the results obtained. Section 5 discusses the main contributions of this research in comparison with the literature, while Sect. 6 summarizes the conclusions drawn from the paper.

## Related works

DL and ML techniques in breast cancer diagnosis have made significant progress in recent years, and various approaches have been developed that achieve high accuracy rates in different imaging modalities. In the literature, especially the integration of TL, ensemble models, transformer-based methods, and XAI techniques have been frequently discussed in order to increase model reliability in clinical applications [14, 17–19].

Early efforts in breast cancer classification relied heavily on machine learning models like support vector machines (SVM) and early DL models like. Pillai et al. [20] obtained 75.46% accuracy on the Mammographic Image Analysis Society (MIAS) dataset using VGG16, highlighting the limitations of standalone CNNs on small datasets. Oza et al. [21] showed that combining CNNs with traditional machine learning classifiers such as SVM can enhance performance, particularly when supported by data augmentation and TL. While these approaches demonstrate improved accuracy, they often suffer from low generalizability and lack robustness across datasets. In contrast, Melekoodappattu et al. [22] addressed this issue by combining handcrafted texture features with DL-extracted features, achieving 98% accuracy using XGBoost on MIAS and Digital Database for Screening Mammography (DDSM). Although effective, such hybrid approaches depend heavily on domain-specific feature engineering. Similarly, Ragab et al. [23] and Samee et al. [24] adopted optimized TL techniques like multi-dimensional CNN (MultiD-CNN) and log-ratio principal component analysis (LR-PCA), reaching high accuracy levels (97.9% and 98.6%, respectively), but did not explore ensemble frameworks or interpretability, which limits clinical deployment. Several studies explored EL and feature fusion to improve classification accuracy. Altameem et al. [25] employed ensemble methods to stabilize performance, while Sannasi Chakravarthy et al. [26] demonstrated that fusing features from multiple CNN architectures and using an SVM for the final classification could achieve. However, these approaches largely focused on performance gains without addressing interpretability or architectural transparency. Ruban et al. [27], in contrast, benchmarked SSD-based models but achieved relatively low performance, underscoring the importance of architectural choice in mammographic analysis.

The utility of TL in low-resource settings has been emphasized by Khan et al. [28], who achieved 97.52% accuracy using GoogLeNet, VGGNet, and ResNet on the BreaKHis dataset. Similarly, Benhassine et al. [29] achieved 94.5% accuracy in a three-class task using DenseNet201 and AlexNet on Breast Ultrasound Images (BUSI) dataset. While these studies underscore the strength of TL in overcoming dataset limitations, their reliance on single-model strategies can limit robustness. Das et al. [30] and Kallipolitis et al. [31] proposed more advanced ensemble systems using multimodal data, achieving 98.08% and 99.25% accuracy on histopathological images. Despite these achievements, mammogram-based TL studies often lack fine-grained interpretability or consistent subtask generalization.

Kamri et al. [32] compared NASNet, VGG16, VGG19, and ResNet models for Breast Imaging Reporting and Data System (BI-RADS) classification using the Moroccan dataset and reported that VGG16 achieved the highest accuracy of 92.53%. Sabani et al. [33] reported 89.8% accuracy and 100% specificity with a CNN model trained on Picture Archiving and Communication Systems (PACS) images. Falconi et al. [34] and Tsai et al. [35] also demonstrated strong performances using EfficientNet and VGG variants. Agnes et al. [15] proposed a custom MA-CNN model, obtaining 96.47% accuracy. While these studies validate DL’s potential in BI-RADS prediction, they often fail to address class imbalance, decision transparency, or ensemble stability.

ViT architectures, one of the most popular techniques in recent times, have gained popularity thanks to their powerful feature extraction capabilities. For example, Nanni et al. [36] integrated ResNet with ViT, whereas Ji et al. [37] employed ViT for molecular subtype classification. Jouirou et al. [38] proposed a two-level content-based retrieval system with 97% accuracy. Models by Ukwuoma et al. [39] and Al-Tam et al. [40] combined transformer encoders with CNN backbones, reporting up to 100% accuracy. However, most of these models prioritize performance at the expense of interpretability, which raises concerns about their use in medical applications. Studies like Jiang et al. [41] and He et al. [42] have shown that integrating ViT models with domain-specific preprocessing (e.g., color deconvolution) can improve accuracy, yet scalability and clinical validation remain open issues. In a recent study, Al-Hejri et al. [43] and colleagues developed the Ensemble Self-Attention Transformer Encoder for Breast Cancer Diagnosis (ETECADx) system for breast cancer detection and achieved accuracy rates of 98.58% and 97.87% using CNN and ViT models on the INbreast and custom datasets, respectively. However, this system has shortcomings in terms of transparency of decision processes. Al-Tam et al. [44] achieved 97.73% accuracy with the integration of YOLOv8 and ViT-based ResNet50, while ensuring model explainability by using Grad-CAM in a hybrid system combining mammography and ultrasound data. In this study, a comprehensive application was carried out to increase both decision transparency and accuracy by blending different CNN architectures with a majority voting-based approach.

Hussain et al. [45] performed an integrated analysis of image and text-based data for breast cancer classification. In the study, six state-of-the-art DL architectures such as VGG16, VGG19, ResNet34, MobileNetV3, EfficientNetB7 and ViT were used for image feature extraction, while Artificial Neural Network (ANN) was used for textual feature extraction and classification. Image and text features were fused with early and late fusion strategies. According to the experimental results, the VGG19 + ANN combination achieved the highest classification success with 95.1% accuracy and 95% precision. VGG16 + ANN provided the best results in this field with 89.3% sensitivity and 92.9% AUC value. These findings show that multimodal data fusion and appropriate model matching are critical in breast cancer diagnosis.

Hussain et al. [46] proposed a method that integrates multiview and multimodal feature fusion with DL approaches to improve accuracy in breast cancer classification. In the study, an enriched feature representation was created by combining data from different angles (Craniocaudal (CC) and Mediolateral Oblique (MLO)) and different information sources obtained from mammography images. The researchers analysed these multiple features with various DL architectures and comparatively evaluated the classification performance. The proposed model integrated both image and text data and evaluated the information obtained from different sources simultaneously. The performance of the model was measured by metrics such as accuracy, precision, F1 score and AUC; the MMFF model achieved an AUC value of 96.5% for benign and malignant discrimination, outperforming image-only and text-only models as well as other multimodal approaches.

Shah et al. [47] developed an ensemble DL approach for early and accurate diagnosis of breast cancer. In the study, EfficientNet, AlexNet, ResNet and DenseNet architectures were combined and each model was specifically optimised. EfficientNet was tuned with dataset-specific scaling, AlexNet reduced the risk of overfitting with variable dropout layers, ResNet uses learnable skip connections, and DenseNet improves both information flow and computational efficiency with selective connections. By combining these models with the ensemble method, higher accuracy and stability were achieved compared to single architectures. The performance of the model was reported with 94.6% precision, 92.4% sensitivity, 96.1% specificity and 98.0% AUC.

XAI integration has become crucial for clinical deployment. Ahmed et al. [48] introduced interpretability using Grad-CAM, SHAP, and LIME with ResNet50, while Peta et al. [49] applied Grad-CAM and SHAP on ESAE-Net, achieving 97.85% accuracy. While these studies improve model transparency, they often rely on post-hoc explanation methods without fully embedding interpretability into the design phase. Moreover, explainability is rarely evaluated across multiple XAI methods simultaneously, limiting the robustness of insights gained.

In contrast to prior studies that predominantly employed single DL models or basic ensemble strategies, the proposed BCECNN framework introduces a structured and multifaceted optimization approach that directly addresses the key limitations identified in the literature. Specifically, it employs a majority-voting ensemble strategy that combines five diverse CNN architectures—AlexNet, VGG16, ResNet18, EfficientNetB0, and XceptionNet—to enhance robustness and classification stability across various tasks. The model is systematically evaluated on the AISSLab organized into five sub-datasets, enabling comprehensive assessment on both binary and multi-class BI-RADS classification scenarios. Moreover, while existing studies often overlook model transparency, BCECNN explicitly integrates multiple XAI techniques—Grad-CAM, LIME, and LIME-Mask—to provide interpretable outputs. These are not only visualized but also clinically validated by an experienced radiologist, ensuring both diagnostic relevance and trustworthiness in real-world applications. By combining ensemble modeling with multi-method explainability and expert evaluation, BCECNN offers a novel contribution that bridges the gap between high performance classification and clinically meaningful decision support an area underexplored in previous literature. This positions BCECNN as a more generalizable, interpretable, and reliable framework for breast cancer detection using mammography, especially in settings with limited data and complex diagnostic needs.

## Materials and methods

In this section, the dataset and methods used in the study are presented in detail under subheadings.

### Overview of the proposed methodology

**Fig. 1:**
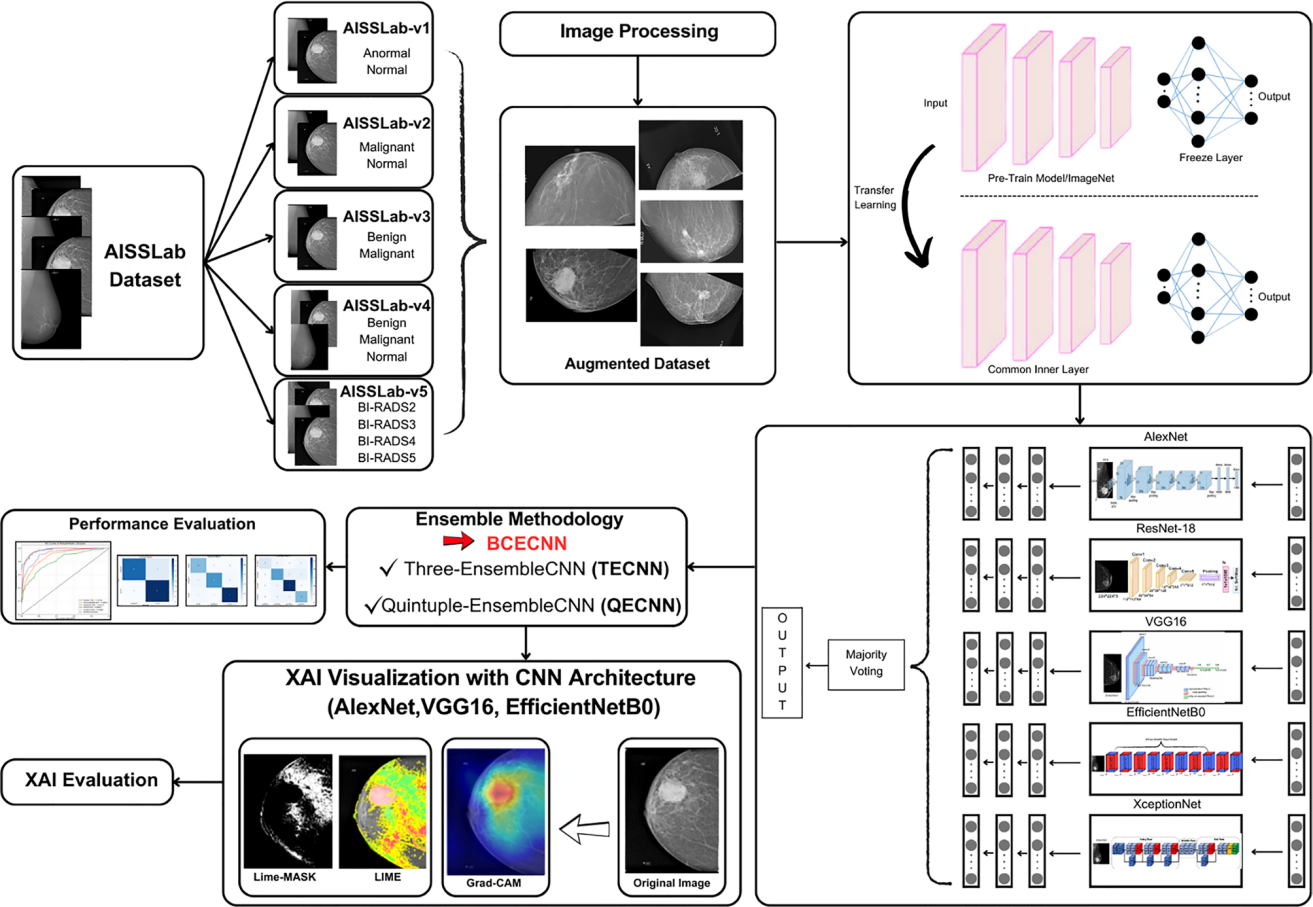
Flow diagram of the proposed method

In this study, an EL-based BCECNN architecture was developed for breast cancer diagnosis, and the flow diagram of the developed architecture is shown in Fig. 1.

As shown in Fig. 1, the proposed method consists of seven stages. First, five different sub-datasets were derived from the publicly available AISSLab dataset in the literature to comprehensively evaluate the method’s performance in various breast cancer classification scenarios. Since the AISSLab dataset contains a relatively small number of samples, multiple data augmentation techniques were applied to increase data diversity, resulting in augmented AISSLab sub-datasets. Next, these datasets were trained using AlexNet [50], ResNet-18 [51], VGG16 [52], EfficientNetB0 [53], and XceptionNet [54] architectures through TL and evaluated on their respective test sets. To further improve performance, EL-based architectures named TECNN and QECNN were developed, employing majority voting across triple and quintuple CNN combinations. The test sections of the AISSLab sub-datasets were then evaluated with these CNN combinations, and the highest-performing CNN and EL architectures were identified. Among them, TECNN—comprising AlexNet, VGG16, and EfficientNetB0, the top-performing CNNs—was integrated into the structure of the proposed BCECNN architecture. To emphasize the contribution of these CNN architectures to the classification task, XAI techniques such as LIME and Grad-CAM were applied, and the corresponding visualizations were generated. Finally, these XAI visualizations were assessed by an expert physician to provide clinical validation of the proposed method’s interpretability. In summary, the proposed approach combines CNN and EL strategies to enhance classification performance, while XAI techniques are leveraged to clarify the reasoning behind the model’s decisions. All stages of the proposed method are described in detail in the subsequent subsections of this section.

### AISSLab dataset

The dataset used in this study was the AISSLab Breast Cancer Dataset [55], created by the Ma’amon’s Diagnostic Centre Mammogram Images for Breast Cancer (MDCMI-BC) in Yemen, for the development of AI-based computer-aided diagnosis systems. Mammogram images are labeled according to the BI-RADS classification and help to determine the probability of lesions being benign or malignant. The data were acquired using a Senographe 800T High-Frequency X-Ray Generator (GE, Florida, USA), and images were taken at CC and MLO angles. However, in some cases, CC images could not be obtained because of the anatomical location of the tumor. In summary, the dataset consists of 266 mammography images and Table 1 shows the overall class status in the dataset.

**Table 1:** Dataset class distribution

Class	Number of Images	Percentage in Total (%)
Benign (BI-RADS 2)	18	6,77
Benign (BI-RADS 3)	48	18,05
Malign (BI-RADS 4)	54	20,30
Malign (BI-RADS 5)	46	17,29
Normal	100	37,59

**Fig. 2:**
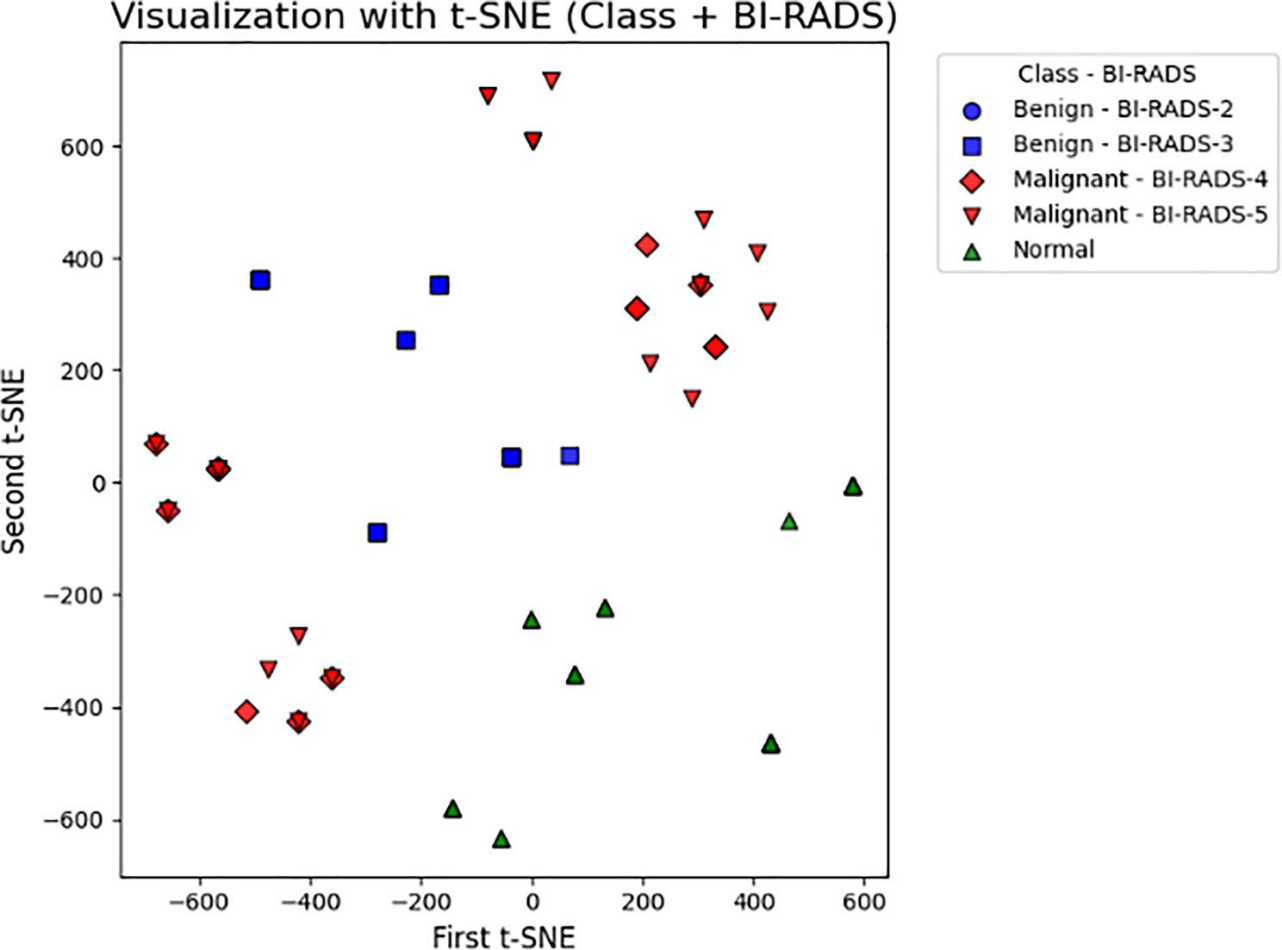
Dataset distribution according to BI-RADS levels by t-SNE method

The t-SNE method was applied to analyze the high-dimensional data distribution and to better understand the relationships between datasets. This method aims to reduce high-dimensional data points to a lower-dimensional space and visualize the level of separation and clustering patterns of different BI-RADS categories [56]. This analysis was performed specifically to assess the transitions between benign and malignant cases and the discriminability of lesions that are diagnostically similar but biologically different. Figure 2 shows the distribution of the dataset obtained with the t-SNE method.

**Fig. 3:**
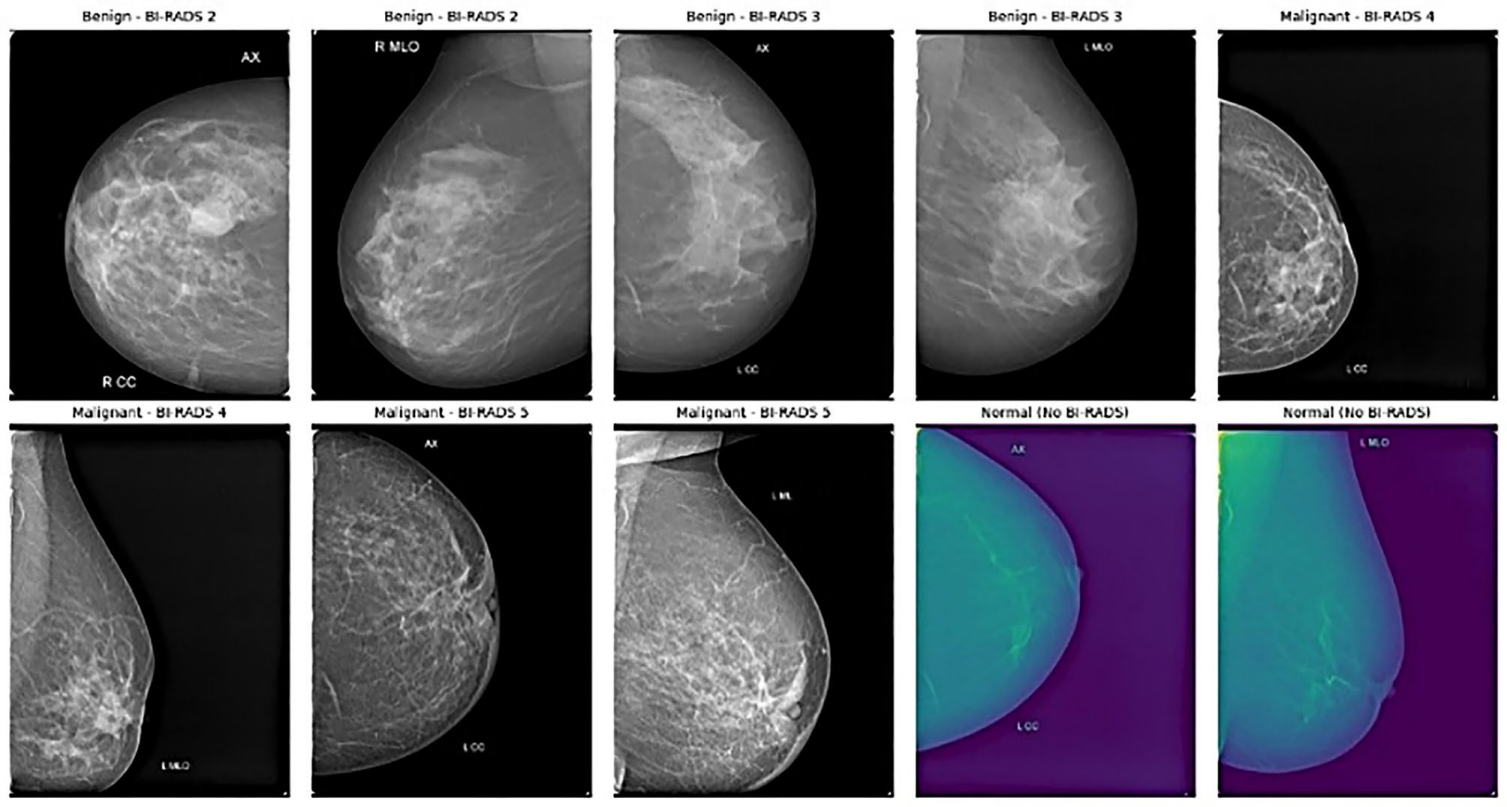
Sample mammography images of different classes from the AISSLab dataset

Sample mammography images selected from the dataset included benign and malignant cases with different BI-RADS levels. Sample mammography images selected from the AISSLab dataset are presented in Fig. 3.

Subgroups were created for different classification tasks from the dataset. This grouping provides different levels of discriminability in the model training processes, enabling the identification of appropriate subsets for general and specific classification scenarios. The sample distributions of both the raw and extended versions of the created sub-datasets are presented in Table 2. For performance evaluation and training process of the CNN architectures used in the proposed method, 90% of both the raw and extended data were allocated for training and 10% for testing, and this ratio was kept constant throughout the study. Finally, the sub-datasets (AISSLab-v1– v5), derived from the AISSLab dataset, have been made publicly available to support future research and promote reusability in the literature.

**Table 2:** Raw and augmented dataset distribution

Dataset		Raw				Augmented		
	Classes	Total	Train	Test	Classes	Total	Train	Test
AISSLab-v1	Normal (100), Anormal (166)	266	239	27	400 664	1064	958	106
AISSLab-v2	Malignant (100), Normal (100)	200	180	20	400 400	800	720	80
AISSLab-v3	Benign (66), Malignant (100)	166	149	17	264 400	664	598	66
AISSLab-v4	Benign (66), Malignant (100), Normal (100)	266	239	27	264 400 400	1064	958	106
AISSLab-v5	BI-RADS 2 (18), BI-RADS 3 (48), BI-RADS 4 (54), BI-RADS 5 (46)	166	149	17	72 192 224 176	664	598	66

### Convolutional neural network

CNN is one of the most widely used architectures in DL-based image processing techniques. Unlike traditional fully connected neural networks, CNNs are based on the principles of local connections and weight sharing. Thanks to these features, they can effectively perform feature extraction from high-dimensional image data [57]. CNN architectures generally consist of three basic components: convolution layers, pooling layers, and fully connected layers. The convolution layers contain filters that learn important features (edges, textures, basic shapes) in the image. Pooling layers reduce the computational load by reducing the processing capacity of the model and help avoid overfitting. unlike classical CNN architectures perform the classification process using the extracted features.

In this study, five different CNN architectures, namely AlexNet, ResNet-18, VGG16, EfficientNetB0, and XceptionNet, are used to perform the breast cancer classification task. These models have different architectural depths and feature extraction capacities, and their performances were compared by testing them on separated datasets. AlexNet [50] was one of the first CNN models that won the ImageNet competition in 2012 and represented a significant advancement in DL. This model consists of eight layers in total, five convolutional layers, and three fully connected layers. Although the depth of the model is limited, it offers an efficient feature extraction mechanism with large kernel sizes and the use of a ReLU activation function. VGG16 [52] is a CNN architecture with a deeper structure and consists of 16 layers. It provides more precise feature extraction by using smaller kernel sizes and offers high performance due to its increased depth, unlike classical CNN architectures. However, the high parameter count of VGG16 increases computational costs. ResNet-18 [51] is a CNN architecture that uses residual connections to solve the vanishing gradient problem encountered during the training of deep networks. Consisting of 18 layers, this model allows for more stable training of deep networks and provides faster convergence. EfficientNetB0 [53] is a model that aims to increase the efficiency of CNN architectures by using a model scaling strategy. Although it has a lower number of parameters than traditional CNN architectures, it can achieve high accuracy rates thanks to its optimized structure. XceptionNet [54] is a CNN model that makes traditional convolution operations more computationally efficient by using depthwise separable convolutions. Its ability to perform a more effective feature extraction with fewer parameters enables the model to achieve successful results, especially in large-scale datasets.

In the proposed BCECNN framework, a diverse set of CNN architectures was strategically selected to balance model depth, computational efficiency, and feature extraction diversity. AlexNet, despite being an older architecture, was included for its lightweight design, fast convergence, and complementary feature extraction, which enhances ensemble diversity and supports real-time deployment. ResNet-18 offers residual learning to address vanishing gradients and ensure stable feature propagation. EfficientNetB0 provides a strong balance between accuracy and efficiency through compound scaling, making it ideal for resource-constrained settings. VGG16 contributes deep hierarchical feature extraction for improved high-level abstraction, while XceptionNet employs depthwise separable convolutions for efficient yet expressive feature learning. Together, these complementary models strengthen the BCECNN ensemble’s robustness, generalizability, and explainability.

**Table 3:** Details of the CNN architectures

Model	Depth	Parameters (Million)	Size (MB)	Input Size
AlexNet [50]	8	61	227	224 × 224
ResNet18 [51]	18	11.7	44	224 × 224
VGG16 [52]	16	138	528	224 × 224
EfficientNetB0 [53]	30	5.3	29	224 × 224
XceptionNet [54]	71	22.9	88	299 × 299

### Image processing

The present study utilized a carefully curated dataset of mammographic images, the complete specifications of which are provided in Table 2. This dataset underwent rigorous preprocessing to ensure optimal compatibility with proposed framework. The initial preprocessing phase involved standard image normalization and resizing operations, where all mammograms were systematically adjusted to conform to the precise input dimensional requirements of the convolutional neural network architectures detailed in Table 3. To address the dual challenges of limited dataset size and potential model overfitting, we implemented an advanced data augmentation pipeline that increased our effective training dataset by a factor of four. This augmentation protocol was specifically designed to enhance model generalizability while preserving the essential diagnostic features of mammographic imagery. The augmentation techniques were selected based on their ability to simulate real-world variations in mammographic acquisition while maintaining pathological relevance. The precise parameters and implementation details of our augmentation methodology are systematically presented in Table 4.

**Table 4:** Data augmentation techniques

Method	Application details
Refraction (Zoom Crop)	Images zoomed in up to 5%, different parts cropped
Rotation	Each image rotated randomly between − 30° and + 30°
Grayscale Conversion	15% of images converted to grayscale
Saturation Adjustment	Random variation of saturation between − 25% and + 25%
Brightness Adjustment	Brightness changed randomly between − 15% and + 15%

**Fig. 4:**
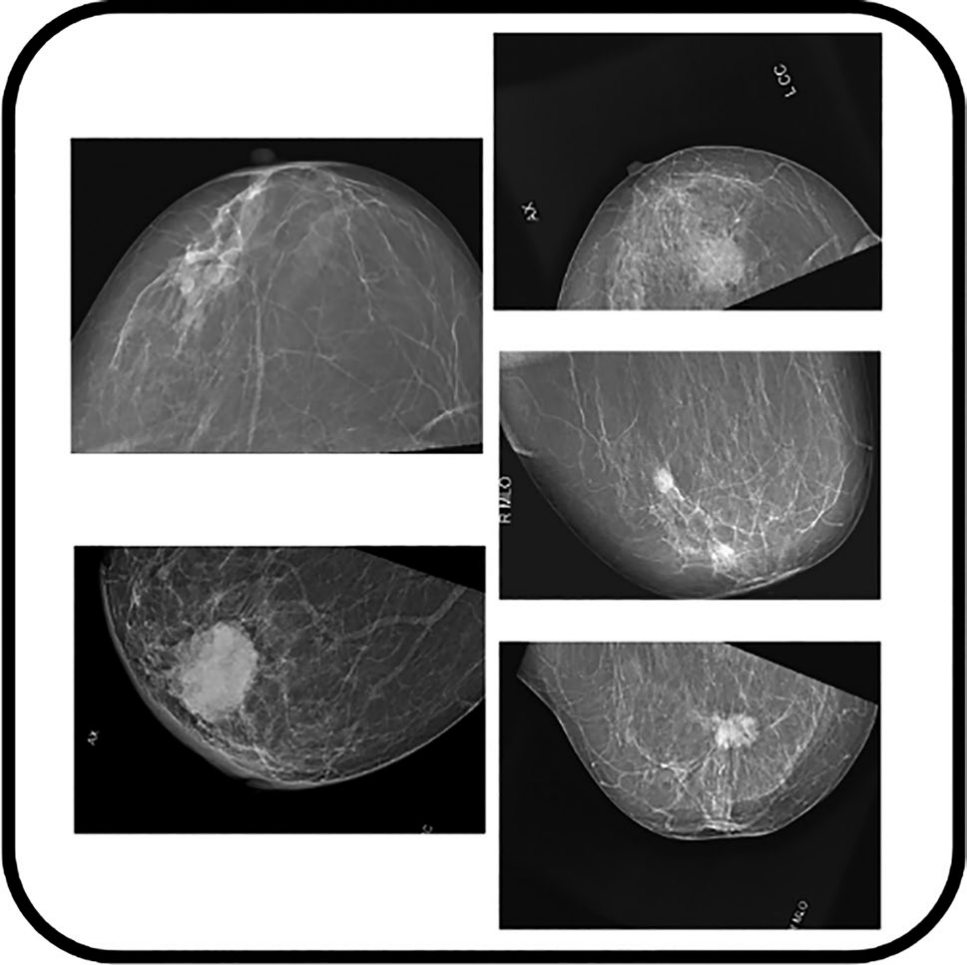
Example visualisations of data augmentation techniques

Representative examples of the augmented images, drawn from the AISSLab dataset, are presented in Fig. 4. This sophisticated augmentation approach not only expanded our training dataset quantitatively but also significantly enhanced its qualitative diversity.

### Transfer learning (TL)

TL is a DL method based on the principle of reusing a model previously trained on large-scale datasets by adapting it to a specific task [58]. The TL method reduces computational costs by shortening the training time and makes it possible to achieve high performance with small datasets [59]. In the TL process, the early layers of the pre-trained models are usually frozen and preserved, while only the upper layers are retrained. The early layers of the model learn low-level features, while the final layers are optimized for more specific classification tasks. In this study, the TL method is applied using AlexNet, VGG16, ResNet-18, EfficientNetB0, and XceptionNet models trained on the ImageNet dataset. In order to preserve the overall feature extraction capacity of these models in the DL process, convolutional layers were frozen, and only fully connected layers were retrained. Thus, it is aimed to adapt the model to the new dataset faster by preserving previously learned information.

The TL method used in this study includes the dropout (0.5) mechanism to prevent the model from overfitting, and softmax and sigmoid activation functions were applied in the classification layers. The model outputs were optimised by using sigmoid functions for two-class classification and logsoftmax functions for multi-class classification, respectively. In addition, techniques such as early stopping and learning-rate scheduling were applied during model training to make the model more stable and generalizable.

### Ensemble Learning (EL)

Ensemble Learning (EL) is a technique that improves the overall model performance by combining the outputs of multiple DL models [14]. Ensemble strategies, which are used to overcome the limitations of a single model and reduce error rates, are of great importance, especially in sensitive classification problems, such as medical image analysis. In this study, two different ensemble approaches were applied using five different CNN models.

Firstly, the Quintuple Ensemble CNN (QECNN) method was tested. This ensemble method, which includes all five models, aims to make maximum use of model diversity. However, the computational cost of this method was high and the diversity was limited because some models have similar feature extraction mechanisms. As this may reduce the generalization capacity of the ensemble model, an alternative strategy was developed by testing triple combinations. With the Triple Ensemble CNN (TECNN) method, 10 different triple combinations of five models were created, and their performances were compared. The triplet and quintet combinations used are given in Table 5.

**Table 5:** List of triple and quintuple combinations

TECNN	AlexNet, VGG16, XceptionNet
	AlexNet, VGG16, EfficientNetB0
	AlexNet, VGG16, ResNet18
	AlexNet, XceptionNet, EfficientNetB0
	AlexNet, XceptionNet, ResNet18
	AlexNet, EfficientNetB0, ResNet18
	VGG16, XceptionNet, EfficientNetB0
	VGG16, XceptionNet, ResNet18
	VGG16, EfficientNetB0, ResNet18
	XceptionNet, EfficientNetB0, ResNet18
QECNN	AlexNet, VGG16, XceptionNet, EfficientNetB0, ResNet18

**Fig. 5:**
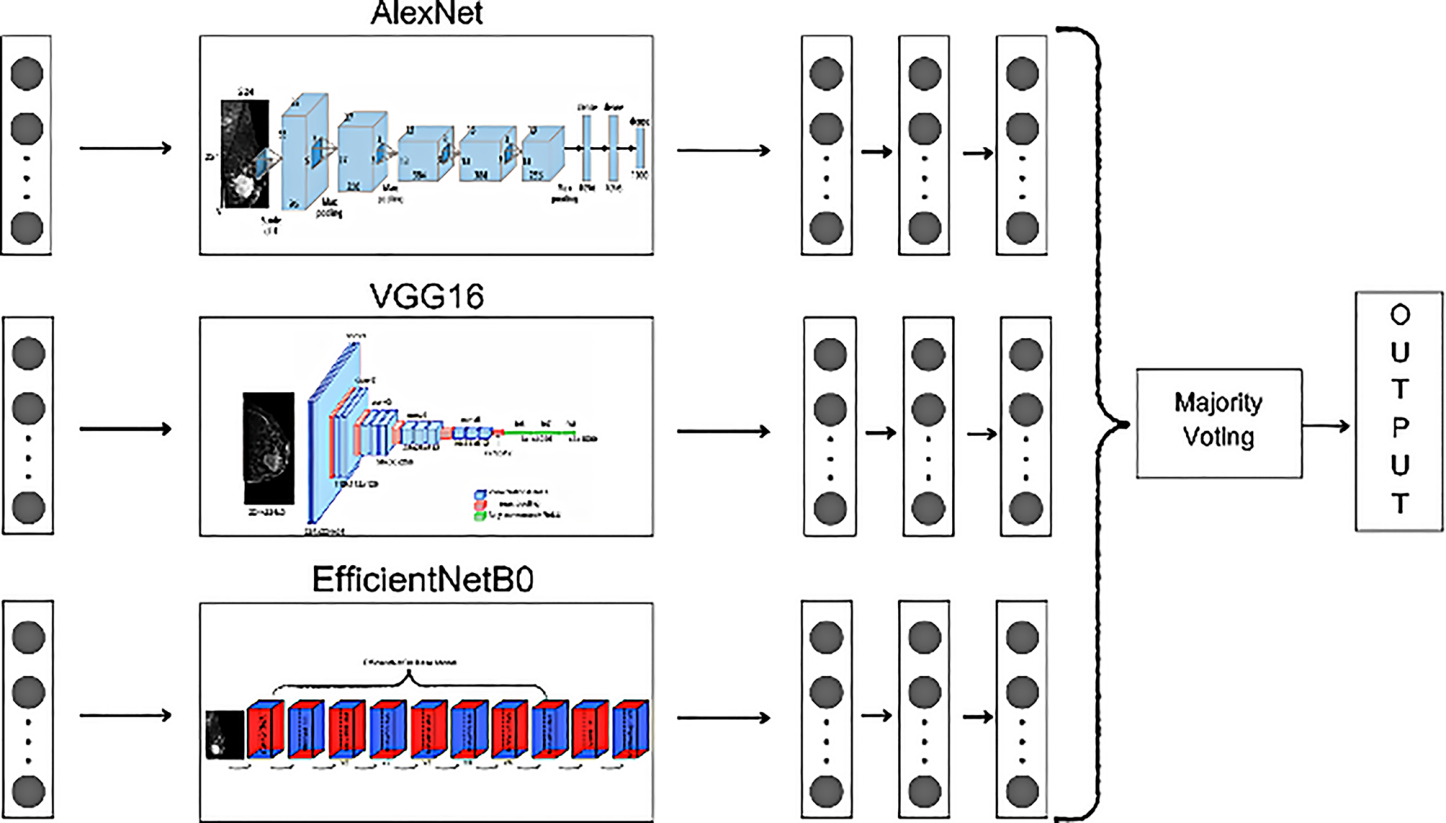
TECNN architecture based on a combination of the highest performing Triple CNN architectures (AlexNet, VGG16 EfficientNetB0)

**Fig. 6:**
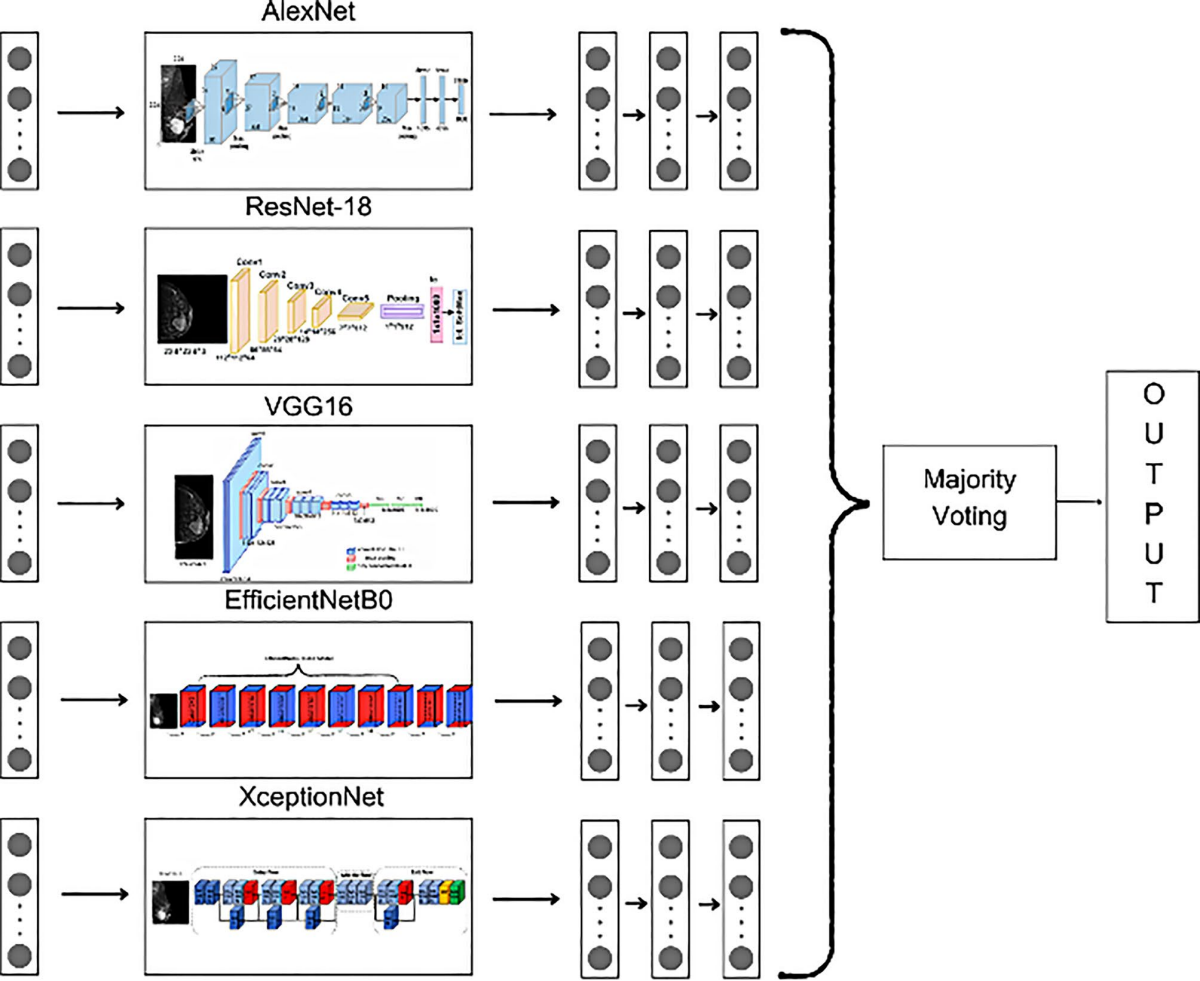
QECNN architecture based on five CNN architectures used

In this study, only triple and quintuple combinations were preferred. The objective was to identify the combination with the highest performance. The ensemble combinations are shown in Figs. 5 and 6. The majority voting [16] method was used in the EL process. In this method, the predictions produced by each model are independently evaluated and the class with the highest number of votes is determined as the final output. Mathematically, majority voting can be expressed in Eq. 1.

1 $$\:\widehat{y}=argmax\:{\sum\:}_{\left\{i=1\right\}}^{N}\left\{1\right\}\left({y}_{i}=\:k\right),\:\:k\:\in\:\{1,\:2,\:\dots\:,\:K\}$$

The majority voting method given in Eq. (1) ensures that the most frequent class label is selected as the final decision according to the predictions of more than one classifier. In this equation, $\:\widehat{y}$ denotes the class label predicted by the majority of the ensemble models, $\:K$ denotes the total number of classes, $\:k\:\in\:\{1,\:2,\:\dots\:,\:K\}$ denotes the candidate class labels, $\:N$ denotes the number of models forming the ensemble and $\:{y}_{i}$ denotes the class predicted by model $\:i$. The indicator function $\:\left\{1\right\}\left({y}_{i}=\:k\right)$ takes the value 1 only if the prediction of model $\:i$ is equal to class $\:k$, otherwise it is 0. Thus, the total number of votes for each class is calculated and the class label with the most votes is assigned as the final prediction.

In triple combinations, the complementary feature extraction capacities of different CNN architectures are combined to increase model diversity and optimize classification accuracy. The choice of these three models is based not only on their individual performance, but also on their complementary architectural features. For example, the shallower structure of AlexNet provides general feature extraction capability, while the deep and homogeneous structure of VGG16 specializes in local and hierarchical features. EfficientNetB0, on the other hand, offers a balanced approach in terms of both efficiency and accuracy, increasing the ability to capture features at different scales. This combination of architectural diversity enabled the model to comprehensively learn a wide range of diagnostic information from breast cancer images. The basic structure of the TECNN architecture is presented in Fig. 5, while the basic structure of the QECNN architecture is shown in Fig. 6.

The experimental results demonstrate that the TECNN strategy performs better than the QECNN method. The main reason for this is that in the QECNN, some CNN architectures have feature extraction mechanisms that are overly similar and produce similar predictions. On the other hand, in TECNN combinations, more balanced model diversity is provided, and classification accuracy is improved. As a result, EL produced more reliable and high accuracy classification results than single CNN models. The model diversity and computational efficiency provided by the TECNN combinations especially increased the success of the EL strategy.

### eXplainable artificial intelligence

DL models are usually black boxes, and it is difficult to understand the internal decision mechanisms directly. This situation has made it imperative to increase model transparency, especially in areas requiring high reliability, such as medical imaging. XAI methods have been developed to ensure the interpretability of AI-based systems. In this study, Grad-CAM and LIME techniques were applied to analyze and visualize the decision mechanisms of CNN models used in the classification process.

Grad-CAM is a technique that determines which image regions the model focuses on by examining the activation maps in the last convolutional layers of CNN models [11]. This method calculates the effect of relevant classes on model prediction using gradient information and produces visually interpretable activation maps. In this study, Grad-CAM was applied to AlexNet, VGG16, and EfficientNetB0 models to analyze which regions are more important to the model when making decisions in mammographic images. Specifically, in the process of identifying malignant tumors, the tissue structures that contributed to the model’s classification decision were examined, thereby evaluating the model’s reliability.

LIME is a method that divides the input image into superpixel segments to explain the classification decisions of the model and measures the effect of each region on the model prediction [12]. This technique analyses the differences in the model’s predictions by randomly changing certain parts of the image and determining which features affect the classification result the most. In this study, LIME and its extended version, LIME-Mask, are used for AlexNet, VGG16, and EfficientNetB0 models, and the attributes on which the model is based are detailed. The LIME-Mask method increases the integrability into clinical evaluation processes, especially by helping to highlight the decisive regions in medical image analyses.

When integrated into proposed method, XAI techniques such as Grad-CAM, LIME, and LIME-Mask enhance the transparency of the BCECNN architecture and provide valuable insights to clinicians during the decision-making process. In particular, during the biopsy decision phase, the model’s ability to visually explain why a lesion was classified as malignant or benign can help radiologists more accurately target suspicious areas and reduce unnecessary invasive procedures.

## Experiments and results

In this study, different CNN architectures such as TECNN and QECNN and EL methods were evaluated and the performance analysis of the models was performed on common classification metrics such as accuracy, sensitivity, sensitivity, specificity, F1 score and AUC. Experiments were conducted on created AISSLAB subdatasets, and the generalization capacity of the obtained models was examined. The performance evaluation of individual CNN models is performed through ROC curves, while the effectiveness of EL methods is visualized with confusion matrices. In addition, the proposed method applies XAI techniques to enhance the interpretability of the decision-making processes of the highest-performing CNN architectures, and the resulting findings are presented. As a result, all the experiments performed in this study are detailed in the following subsections.

### Experimental setting

All experiments related to the proposed method and the pretrained CNN architectures used in this study were performed on an Intel Core i9-13900HX (13th Generation, 2.20 GHz) processor, 32 GB RAM and NVIDIA RTX 4090 GPU. The training times obtained under this hardware configuration for different models, data types, and epoch settings are summarized in Table 6.

**Table 6:** Training times of pretrained CNN models by data type and epoch

Dataset	Epoch	Model	Training Time
Raw	50	AlexNet	9 m 33.3s
		VGG16	10 m 8.0s
		XceptionNet	10 m 6.0s
		EfficientNetB0	10 m 19.3s
		ResNet18	9 m 32.2s
	100	AlexNet	17 m 58.5s
		VGG16	20 m 40.6s
		XceptionNet	21 m 27.3s
		EfficientNetB0	18 m 55.2s
		ResNet18	19 m 7.3s
Augmented	50	AlexNet	16 m 16.2s
		VGG16	18 m 3.1s
		XceptionNet	22 m 48.4s
		EfficientNetB0	16 m 56.0s
		ResNet18	18 m 3.5s
	100	**AlexNet**	40 m 17.9s
		**VGG16**	35 m 36.4s
		XceptionNet	36 m 45.5s
		**EfficientNetB0**	34 m 29.1s
		ResNet18	39 m 42.0s

As can be seen in Table 6, while training with raw dataset usually takes less time, training times increased significantly when data augmentation was applied; especially for models such as XceptionNet and AlexNet for 100 epochs, the training time approached 40 min. This comparison shows that the data type and the number of epochs have a direct effect on the training time.

### Hyperparameter selection

In this study, some hyperparameters had to be reconstructed for CNN training. During model training, various hyperparameters affecting performance were carefully determined. A learning rate of 0.0001 was chosen because a very small learning rate allows the model to update its weights more precisely and to learn in a more balanced way without sudden jumps. Adam was chosen as the optimization algorithm, which is a powerful optimizer that accelerates the learning process with both adaptive learning rate adjustments and momentum information. The mini-batch size was fixed at 32, which is both efficient in terms of memory usage and provides sufficient stability in gradient estimation. In order to prevent overfitting, 50% dropout was applied in fully connected layers. In the classification layers, appropriate activation functions were used according to the problem type: sigmoid for two-class problems and softmax for multi-class problems. Cross-entropy loss was used for binary classification tasks, while log-softmax-based negative log-likelihood loss was employed for multi-class classification. As a result, the details of the hyperparameters identified in this study are presented in Table 7.

**Table 7:** Details of hyperparameters used for CNN architectures

Hyperparameter	Value
Learning Rate	0.0001
Optimizer	Adam
Batch Size	32
Dropout Rate	0.5
Activation Function	Softmax / Sigmoid
Loss Function	Crossentropy
Epoch	50–100

### Performance metrics

Various statistical metrics were used to evaluate the model’s performance. Accuracy, defined in Eq. (2), refers to the overall correct classification rate of the model over all samples; it is calculated as the ratio of true positive (TP) and true negative (TN) predictions to the total number of samples (TP + TN + FP + FN). The precision in Eq. (3) represents the proportion of samples that the model predicts as positive that are actually positive and is defined by the formula TP / (TP + FP). The specificity in Eq. (4) indicates the proportion of the negative class correctly predicted, i.e. the ratio of true negatives to total negatives (TN + FP). Sensitivity in Eq. (5), also known as true positive rate (recall), indicates the extent to which the model correctly detects positive classes and is calculated by the formula TP / (TP + FN). The F1 score given in Eq. (6) is the harmonic mean of the precision and sensitivity metrics and provides a balanced performance measure in imbalanced datasets. Finally, the AUC defined in Eq. (7) refers to the area under the ROC curve; it is calculated by integrating the false positive rate (FPR) on the horizontal axis and the true positive rate (TPR) on the vertical axis. The AUC value is a strong indicator summarizing the model’s ability to distinguish between positive and negative classes [60].

2 $$\:Accuracy\:=\:\frac{TP+TN}{TP\:+\:TN\:+\:FP\:+\:FN}$$

3 $$\:Precision\:=\frac{TP\:}{TP\:+\:FP}$$

4 $$\:Specificity\:=\:\frac{TN\:}{TN\:+\:FP}$$

5 $$\:Sensitivity\:=\frac{TP}{TP\:+\:FN}$$

6 $$\:{F}_{1}\:score=\:2\:x\:\frac{Precision\:x\:Sensitivity}{Precision+Sensitivity}$$

7 $$\:AUC\:=\:{\int\:}_{0}^{1}TPR\left(FPR\right)d\left(FPR\right)$$

### Performance results of CNN architectures on raw and augmented AISSLab subdatasets

**Fig. 7:**
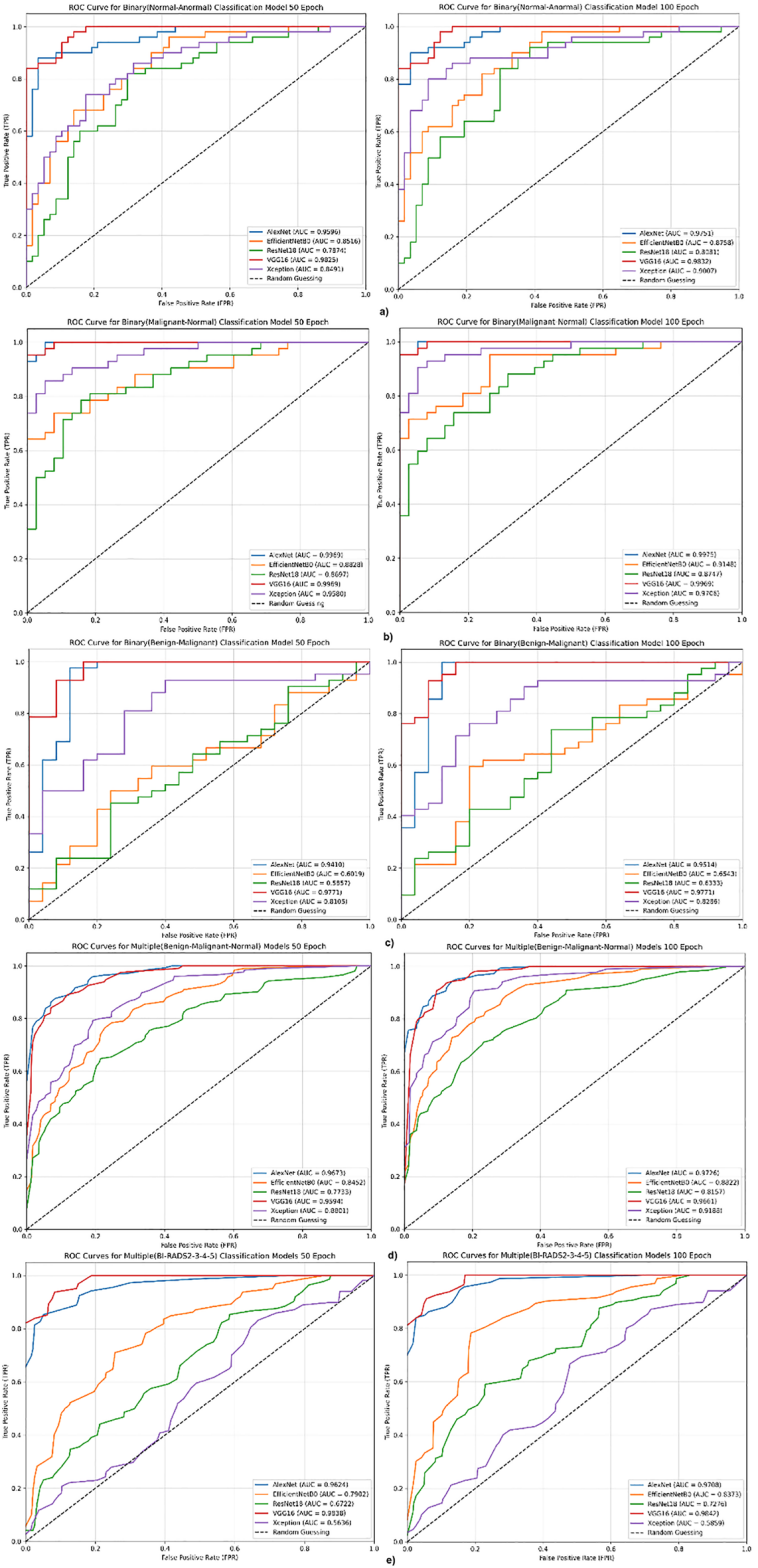
ROC curves of the augmented AISSLab subdatasets: **a**)v1, **b**)v2, **c**) v3, **d**)v4, **e**)v5

The CNN architectures used in this study were trained and tested on raw AISSLab subsets. Following testing, their performance was evaluated using metrics such as accuracy, precision, sensitivity, specificity, F1 score, and AUC. To comprehensively assess the classification capabilities of the individual CNN models, ROC curves were constructed for visual analysis and presented in Fig. 7. The performance results of the CNN models trained on the raw dataset are presented in Table 8. The findings indicate that models such as XceptionNet, AlexNet, and EfficientNetB0 outperform others on the AISSLab datasets. In particular, the EfficientNetB0 model demonstrated superior performance in terms of precision. AlexNet also exhibited strong performance in terms of AUC on AISSLab-v2; however, its generalization capability appears more limited compared to the other models.

In the subsequent evaluation, CNN architectures were trained and tested using the augmented AISSLab subdatasets. The performance metrics obtained after testing are presented in Table 9. Compared to the other models, VGG16 and AlexNet showed notable improvements in accuracy and AUC when data augmentation was applied. In particular, the AlexNet model exhibited a significant increase in sensitivity and specificity across experiments conducted at different epochs, following data augmentation. These results indicate that the model benefits from increased data diversity during training and is able to maintain strong classification performance even in low-data scenarios. As a result, as seen in Tables 8 and 9, AlexNet, VGG16, EfficientNetB0 stand out as the most effective CNN architectures compared to others, showing remarkable performances in terms of performance metrics on both raw and augmented datasets.

**Table 8:** Performance comparison of CNN architectures trained on Raw dataset

Data	Epoch	Method	Accuracy	Precision	Sensitivity	Specificity	F1 Score	AUC
AISSLab-v1	50	AlexNet	0.8519	0.6000	0.6000	**0.9091**	0.6000	0.9091
		VGG16	0.8519	0.5714	0.8000	0.8636	0.6667	0.9000
		XceptionNet	**0.8889**	**0.6250**	**1.0000**	0.8636	**0.7692**	**0.9818**
		EfficientNetB0	0.7407	0.2500	0.2000	0.8636	0.2222	0.8273
		ResNet18	0.8148	0.5000	0.4000	**0.9091**	0.4444	0.7545
	100	AlexNet	0.8148	0.5000	0.6000	0.8636	0.5455	0.8727
		VGG16	0.8519	0.5714	0.8000	0.8636	0.6667	0.9000
		XceptionNet	**0.8889**	**0.6250**	**1.0000**	**0.8636**	**0.7692**	**0.9818**
		EfficientNetB0	0.8148	0.5000	0.6000	0.8636	0.5455	0.8818
		ResNet18	0.8148	0.5000	0.6000	0.8636	0.5455	0.7545
AISSLab-v2	50	AlexNet	**0.8500**	0.7000	**1.0000**	0.7692	**0.8235**	**0.9120**
		VGG16	0.6000	0.4444	0.5714	0.6153	0.5000	0.6813
		XceptionNet	0.8000	**0.7142**	0.7142	**0.8461**	0.7142	0.7802
		EfficientNetB0	0.7000	0.5555	0.7142	0.6923	0.6250	0.7362
		ResNet18	0.7500	0.6250	0.7142	0.7692	0.6666	0.7362
	100	AlexNet	**0.8500**	**0.7500**	**0.8571**	**0.8461**	**0.8000**	**0.9120**
		VGG16	0.6500	0.5000	0.5714	0.6923	0.5333	0.6813
		XceptionNet	0.7500	0.6666	0.5714	**0.8461**	0.6153	0.8021
		EfficientNetB0	0.7500	0.6250	0.7142	0.7692	0.6666	0.7692
		ResNet18	0.7000	0.5714	0.5714	0.7692	0.5714	0.8461
AISSLab-v3	50	AlexNet	0.5882	0.6429	0.8182	0.1667	0.7200	0.6061
		VGG16	0.5882	0.6429	0.8182	0.1667	0.7200	**0.7273**
		XceptionNet	0.7059	**0.8000**	0.7273	**0.6667**	0.7619	0.6515
		EfficientNetB0	**0.7647**	0.7333	**1.0000**	0.3333	**0.8462**	0.4697
		ResNet18	0.6471	0.6471	**1.0000**	0.0000	0.7857	0.4697
	100	AlexNet	0.6471	0.6667	0.9091	0.1667	0.7692	0.6667
		VGG16	0.5882	0.6429	0.8182	0.1667	0.7200	**0.7121**
		XceptionNet	0.7059	**0.8000**	0.7273	**0.6667**	0.7619	0.6364
		EfficientNetB0	**0.7647**	0.7333	**1.0000**	0.3333	**0.8462**	0.4242
		ResNet18	0.6471	0.6471	**1.0000**	0.0000	0.7857	0.5758
AISSLab-v4	50	AlexNet	0.4444	0.4444	0.5107	0.7164	0.4530	0.7130
		VGG16	0.4444	0.4444	0.5107	0.7164	0.4530	0.6300
		XceptionNet	0.5185	0.3589	0.5476	0.7586	0.4074	**0.7505**
		EfficientNetB0	0.5185	0.5185	0.5654	**07877**	0.4591	0.7226
		ResNet18	**0.5925**	**0.7070**	**0.6130**	0.7808	**0.5122**	0.6639
	100	AlexNet	0.4074	0.4101	0.4444	0.6988	0.4098	0.6976
		VGG16	0.4074	0.4111	0.4869	0.7012	0.4166	0.6392
		XceptionNet	0.5555	0.3896	0.5714	0.7714	0.4464	0.7627
		EfficientNetB0	0.5555	0.5865	**0.6250**	**0.8005**	0.5401	**0.7672**
		ResNet18	**0.6296**	**0.7106**	0.6119	0.7960	**0.5682**	0.7107
AISSLab-v5	50	AlexNet	0.1765	0.0972	0.2500	0.7321	0.1394	0.6518
		VGG16	0.1176	0.0625	0.1250	0.7071	0.0833	0.5551
		XceptionNet	**0.3529**	0.1587	0.3036	0.7500	0.2083	0.5826
		EfficientNetB0	**0.3529**	**0.3958**	**0.3839**	**0.7936**	**0.3049**	**0.6592**
		ResNet18	0.1765	0.1399	0.2232	0.7263	0.1534	0.5863
	100	AlexNet	0.2353	0.1889	0.2857	0.7487	0.1984	0.6696
		VGG16	0.1176	0.0625	0.1250	0.7071	0.0833	0.5707
		XceptionNet	0.2353	0.1146	0.2321	0.7333	0.1438	0.5625
		EfficientNetB0	**0.3529**	**0.3208**	**0.3571**	**0.7878**	**0.3078**	**0.6830**
		ResNet18	0.1765	0.1399	0.2232	0.7263	0.1534	0.6659

**Table 9:** Performance comparison of CNN architectures trained on augmented dataset

Data	Epoch	Method	Accuracy	Precision	Sensitivity	Specificity	F1 Score	AUC
AISSLab-v1	50	AlexNet	0.9159	0.9556	**0.8600**	0.9649	0.9053	0.9596
		VGG16	**0.9252**	**1.0000**	0.8400	**1.0000**	**0.9130**	**0.9825**
		XceptionNet	0.7944	0.7917	0.7600	0.8246	0.7755	0.8635
		EfficientNetB0	0.7477	0.8485	0.5600	0.9123	0.6747	0.8516
		ResNet18	0.5888	0.8000	0.1600	0.9649	0.2667	0.7874
	100	AlexNet	0.8972	0.9535	0.8200	0.9649	0.8817	0.9751
		VGG16	**0.9252**	**1.0000**	**0.8400**	**1.0000**	**0.9130**	**0.9832**
		XceptionNet	0.8318	0.8636	0.7600	0.8947	0.8085	0.8751
		EfficientNetB0	0.7757	0.8611	0.6200	0.9123	0.7209	0.8758
		ResNet18	0.6542	0.8095	0.3400	0.9298	0.4789	0.8081
AISSLab-v2	50	AlexNet	0.9500	0.9524	**0.9524**	0.9474	0.9524	**0.9969**
		VGG16	**0.9750**	**1.0000**	**0.9524**	**1.0000**	**0.9756**	**0.9969**
		XceptionNet	0.8500	0.8261	0.9048	0.7895	0.8636	0.9580
		EfficientNetB0	0.7750	0.7857	0.7857	0.7632	0.7857	0.8828
		ResNet18	0.7750	0.7727	0.8095	0.7368	0.7907	0.8697
	100	AlexNet	**0.9500**	**0.9524**	**0.9524**	**0.9474**	**0.9524**	**0.9975**
		VGG16	**0.9500**	**0.9524**	**0.9524**	**0.9474**	**0.9524**	0.9969
		XceptionNet	0.9250	0.9286	0.9286	0.9211	0.9286	0.9706
		EfficientNetB0	0.7875	0.7907	0.8095	0.7632	0.8000	0.9148
		ResNet18	0.7500	0.7619	0.7619	0.7368	0.7619	0.8747
AISSLab-v3	50	AlexNet	**0.9254**	**0.9111**	**0.9762**	**0.8400**	**0.9425**	0.9410
		VGG16	0.9104	0.9091	0.9524	**0.8400**	0.9302	**0.9771**
		XceptionNet	0.7612	0.7500	0.9286	0.4800	0.8298	0.8105
		EfficientNetB0	0.6269	0.6545	0.8571	0.2400	0.7423	0.6019
		ResNet18	0.6119	0.6290	0.9286	0.0800	0.7500	0.5857
	100	AlexNet	**0.9403**	**0.9130**	**1.0000**	**0.8400**	**0.9545**	0.9514
		VGG16	0.9104	0.9091	0.9524	**0.8400**	0.9302	**0.9771**
		XceptionNet	0.8060	0.8085	0.9048	0.6400	0.8539	0.8286
		EfficientNetB0	0.6119	0.6667	0.7619	0.3600	0.7111	0.6543
		ResNet18	0.6119	0.6429	0.8571	0.2000	0.7347	0.6333
AISSLab-v4	50	AlexNet	**0.8692**	**0.8808**	**0.8550**	**0.9257**	**0.8660**	**0.9642**
		VGG16	0.8224	0.8192	0.8123	0.9118	0.8083	0.9540
		XceptionNet	0.7103	0.6813	0.6884	0.8598	0.6823	0.8664
		EfficientNetB0	0.6729	0.6319	0.6241	0.8295	0.6268	0.8291
		ResNet18	0.5701	0.6566	0.5665	0.7828	0.5690	0.7626
	100	AlexNet	**0.8505**	**0.8520**	0.8401	0.9184	0.8437	**0.9706**
		VGG16	**0.8505**	0.8413	**0.8730**	**0.9322**	**0.8449**	0.9655
		XceptionNet	0.7570	0.7388	0.7515	0.8853	0.7394	0.9088
		EfficientNetB0	0.7009	0.6667	0.6692	0.8502	0.6673	0.8684
		ResNet18	0.5888	0.6152	0.5657	0.7893	0.5683	0.8023
AISSLab-v5	50	AlexNet	0.8209	0.8473	0.7794	0.9342	0.8048	0.9538
		VGG16	**0.8806**	**0.8866**	**0.8568**	**0.9596**	**0.8664**	**0.9790**
		XceptionNet	0.3731	0.1957	0.2951	0.7685	0.2139	0.5603
		EfficientNetB0	0.5672	0.5120	0.5011	0.8437	0.5050	0.7858
		ResNet18	0.4925	0.4392	0.3791	0.8070	0.3813	0.6639
	100	AlexNet	0.8209	0.8473	0.7794	0.9342	0.8048	0.9622
		VGG16	**0.8806**	**0.8896**	**0.8568**	**0.9579**	**0.8687**	**0.9793**
		XceptionNet	0.3881	0.3396	0.3214	0.7797	0.2382	0.5752
		EfficientNetB0	0.6418	0.6109	0.5869	0.8698	0.5959	0.8371
		ResNet18	0.5075	0.4589	0.4207	0.8112	0.4237	0.7134

As seen in Fig. 7, the CNN models trained with augmentation dataset are more stable and yield higher AUC values. The ROC curves obtained from training the CNN architectures on the augmented dataset indicate that these models exhibit more consistent performance compared to those trained on the raw dataset. Upon analyzing the ROC curves across all subdatasets, VGG16 and AlexNet demonstrated superior performance in terms of AUC compared to the other models. While ResNet-18 showed some improvement after data augmentation, it still achieved relatively low AUC values. In summary, based on the findings, AlexNet and VGG16 emerged as the top-performing architectures, followed by EfficientNetB0 and XceptionNet, with ResNet showing the lowest performance.

### Performance evaluation of TECNN and QECNN strategies

EL methods are used to increase the performance of individual CNN models [36]. In this study, two different EL strategies, TECNN and QECNN, based on majority voting, were applied to the test datasets of AISSLab subsets and a comparison of the findings according to performance metrics is given in Table 10.

When Table 10 is analyzed, TECNN and QECNN obtained with different combinations of CNN architectures generally outperform individual CNN architectures, while TECNN outperforms QECNN in all performance metrics. The TECNN combination using AlexNet, VGG16 and EfficientNetB0 achieved the best performance compared to the other TECNN combinations on the AISSLabv2-v4-v5 subsets, while the TECNN using AlexNet, VGG16 and XceptionNet performed the best on the AISSLabv1-v3 subsets. However, considering the overall accuracy, the combination of TECNN using AlexNet, VGG16 and EfficientNetB0 achieved the highest performance with 91.53% and was used in the structure of the proposed methodology. As a result, Table 11 shows the confusion matrices of the highest-performing TECNN combination after testing at 50 and 100 epochs.

When Table 11 is examined, it is seen that the performance evaluations with 50 and 100 epochs did not provide a relative performance improvement except for AISSLabv4, indicating that 50 epochs is an appropriate training parameter for the CNN architectures used.

**Table 10:** Performance comparison of TECNN and QECNN models in terms of accuracy metrics

Dataset		AISSLab-v1		AISSLab-v2		AISSLab-v3		AISSLab-v4		AISSLab-v5		Overall
Epoch		50	100	50	100	50	100	50	100	50	100	
TECNN	AlexNet, VGG16, XceptionNet	**0.9439**	**0.9439**	0.9750	0.9500	**0.9254**	**0.9254**	0.8692	0.8411	0.8358	0.8358	0.9046
	AlexNet, VGG16, EfficientNetB0	0.9159	0.9252	**0.9875**	**0.9875**	0.9104	0.9104	**0.8785**	**0.9065**	**0.8657**	**0.8657**	**0.9153**
	AlexNet, VGG16, ResNet18	0.8972	0.8972	0.9875	0.9625	0.8806	0.8955	0.8598	0.8785	0.8209	0.8209	0.8901
	AlexNet, XceptionNet, EfficientNetB0	0.8972	0.8972	0.8750	0.8750	0.7015	0.7313	0.7103	0.7664	0.7313	0.7910	0.7976
	AlexNet, XceptionNet, ResNet18	0.8972	0.8785	0.8625	0.8375	0.6418	0.7015	0.7383	0.7570	0.7164	0.7313	0.7762
	AlexNet, EfficientNetB0, ResNet18	0.7757	0.8131	0.8625	0.8625	0.6716	0.6866	0.7944	0.7944	0.6418	0.7015	0.7604
	VGG16, XceptionNet, EfficientNetB0	0.9065	0.9159	0.8875	0.8625	0.6567	0.6866	0.7570	0.8318	0.7612	0.8209	0.8087
	VGG16, XceptionNet, ResNet18	0.9065	0.9065	0.8750	0.8500	0.6269	0.6716	0.7103	0.7757	0.7313	0.7463	0.7800
	VGG16, EfficientNetB0, ResNet18	0.7757	0.8411	0.8625	0.8875	0.6716	0.6716	0.7570	0.7664	0.6866	0.7463	0.7666
	XceptionNet, EfficientNetB0, ResNet18	0.7570	0.7944	0.7750	0.7750	0.6119	0.5970	0.6168	0.6542	0.5522	0.6418	0.6775
QECNN	AlexNet, VGG16, XceptionNet, EfficientNetB0, ResNet18	0.9065	0.9346	0.9250	0.9375	0.6716	0.7164	0.8317	0.8785	0.7761	0.8209	0.8399

**Table 11:** Confusion matrices of highest-performing TECNN on AISSLab subdatasets

Dataset	Epoch	True Label	Predicted: Class 1	Predicted: Class 2	Predicted: Class 3	Predicted: Class 4
AISSLab-v1	50	Normal	57	0	–	–
		Anormal	9	41	–	–
	100	Normal	57	0	–	–
		Anormal	8	42	–	–
AISSLab-v2	50	Malignant	37	1	–	–
		Normal	0	42	–	–
	100	Malignant	37	1	–	–
		Normal	0	42	–	–
AISSLab-v3	50	Benign	21	4	–	–
		Malignant	2	40	–	–
	100	Benign	21	4	–	–
		Malignant	2	40	–	–
AISSLab-v4	50	Benign	17	3	2	–
		Malignant	1	31	2	–
		Normal	2	3	46	–
	100	Benign	21	1	1	–
		Malignant	3	29	2	–
		Normal	2	1	47	–
AISSLab-v5	50	BI-RADS 2	4	1	0	1
		BI-RADS 3	5	15	2	1
		BI-RADS 4	0	1	27	0
		BI-RADS 5	1	1	1	12
	100	BI-RADS 2	4	1	1	0
		BI-RADS 3	5	15	2	1
		BI-RADS 4	0	1	27	0
		BI-RADS 5	1	1	1	12

Tables 8 and 9 demonstrate that the CNN architectures trained on the augmented AISSLab subdatasets achieved significantly better performance compared to those trained on the raw dataset, highlighting the positive impact of data augmentation techniques on learning. In the performance evaluation of the TECNN and QECNN strategies presented in Table 10, TECNN combinations outperformed QECNN, particularly with CNN architectures such as AlexNet, VGG16, and EfficientNetB0. A detailed analysis of the prediction results from the top-performing TECNN combination revealed that in the majority vote-based approach, VGG16 and EfficientNetB0 correctly classified instances misclassified by AlexNet, and vice versa. This indicates that the EL strategy enhances generalization by leveraging complementary decision mechanisms across multiple CNN architectures. Consequently, the TECNN architecture emerged as one of the highest-performing methods in this study, offering a notable performance improvement over single CNN models through the majority voting approach.

### McNemar statistical test

In addition to the performance analysis of the TECNN and QECNN models, the McNemar test was used to assess whether the observed performance difference was statistically significant. This non-parametric test examines the discordant pairs—specifically, the number of instances where one model predicts correctly while the other does not—to determine if the performance difference is likely due to chance [61]. For the comparison of the predictions of TECNN and QECNN, a contingency table for the McNemar test was created and is shown in Table 12.

**Table 12:** Contingency table of the McNemar test of QECNN and TECNN models

	TECNN correct	TECNN incorrect
QECNN correct	*n₁₁ =70*	**n₀₁ = 1**
QECNN incorrect	**n₁₀ = 9**	*n₀₀ = 0*

As shown in Table 12, TECNN correctly classified 9 instances that QECNN misclassified, whereas QECNN correctly classified only 1 instance misclassified by TECNN. Based on these values in this table, the statistic (χ²) was calculated as 5.33 with 1 degree of freedom in McNemar test, yielding a p-value of 0.023. In this study, the null hypothesis stating that there is no performance difference between the EL models was tested and subsequently rejected, as the obtained p-value was below the commonly accepted significance threshold of 0.05. Thus, the statistical analysis with the McNemar test showed that the TECNN architecture was able to achieve better prediction results compared to the QECNN architecture.

### XAI evaluation

**Fig. 8:**
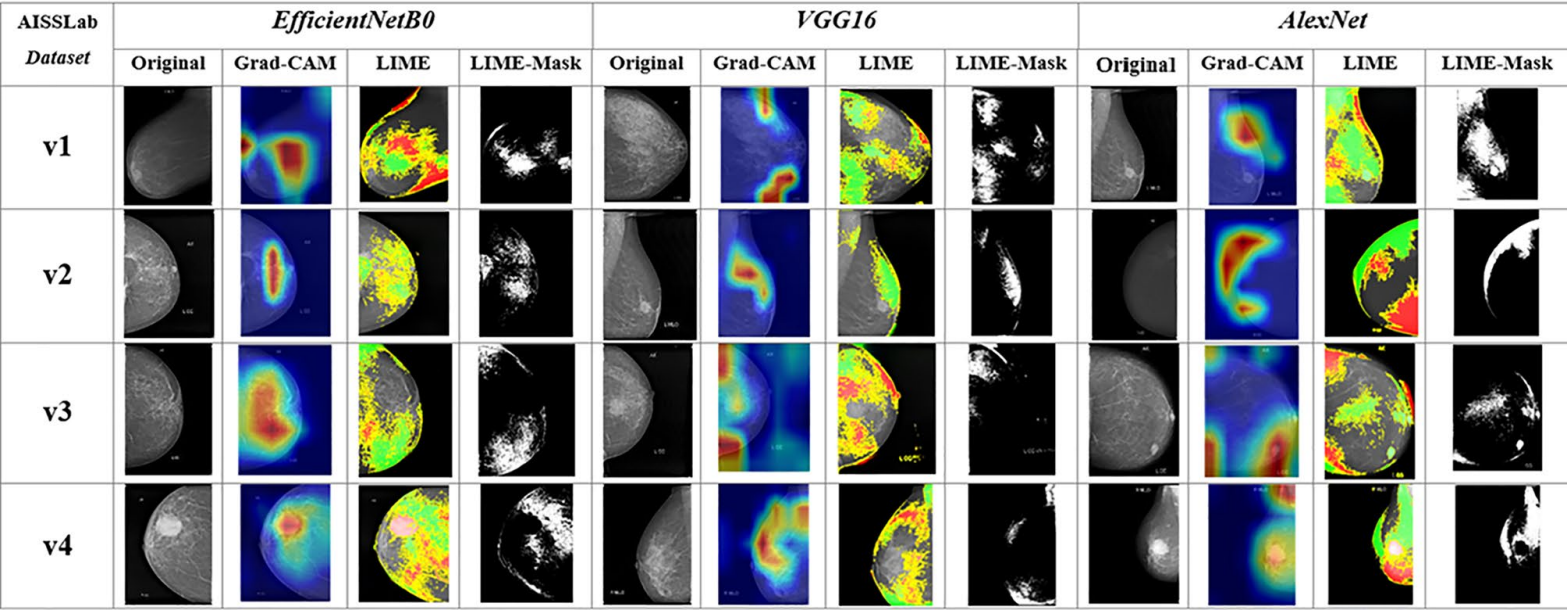
XAI visualizations using Grad-CAM, LIME, and LIME-Mask for the high-performing CNN architectures in the TECNN combination

In this study, the decision processes of the highest-performing CNN architectures were visualized with XAI methods such as Grad-CAM, LIME and LIME-Mask, and the XAI visualizations obtained are shown in Fig. 8. As seen Fig. 8, the EfficientNetB0 and VGG16 architectures generated meaningful and localized heatmaps using the Grad-CAM method, specifically highlighting lesion regions in mammography images. The LIME and LIME-Mask visualizations further enhanced interpretability by clearly delineating the areas contributing to each model’s decision-making process. In contrast, the AlexNet model produced more dispersed and broadly distributed activations, suggesting lower selectivity and overall performance limitations. The consistency observed across all dataset versions (v1–v4) indicates that EfficientNetB0 and VGG16 maintain stable decision processes under varying data conditions. These findings demonstrate that the models within the proposed TECNN framework are strong candidates not only for their high accuracy but also for their explainability.

The obtained XAI visualizations were also visually evaluated by a specialist physician with 35 years of experience, and as a result of the evaluations, it was determined that the heat maps reflect clinically meaningful regions in the decision-making process, especially EfficientNetB0 and VGG16 architectures provide more reliable outputs in terms of interpretability. This evaluation reveals that the decisions of the proposed architecture are not only statistically successful but also clinically meaningful.

## Discussion

Breast cancer is one of the most common types of malignancy among women worldwide, and its early detection directly affects the prognosis of the disease. Traditional diagnostic methods are based on radiologist’s interpretations and may be prone to subjective errors. In recent years, AI-assisted diagnostic systems, especially DL models, aim to increase accuracy rates by automating the diagnostic processes and as a result of the detailed literature review in Sect. 2 of this study, a summary of previous studies on breast cancer classification is shown in Table 13.

**Table 13:** Performance comparison of previous studies for breast cancer classification

References	Dataset	Class	Method	Accuracy(%)	XAI Method
Montaha et al. [62]	CBIS-DDSM	Benign calcification, Benign mass, Malignant calcification, Malignant mass	VGG16 based BreastNet18	98.02	-
Abbasniya et al. [64]	BreakHis	Benign, Malign	Inception-ResNet-v2 / XGBoost, LightGBM, CatBoost (IRv2-CXL)	96.46	Grad-CAM
Samee et al. 2022 [24]	INbreast, mini-MIAS	Benign, Malign	AlexNet, VGG16, GoogleNet	98.50	-
Samee et al. 2022 [65]	INbreast	Benign, Malign	HybridTL of CNN-based LR-PCA	98.60	-
Al-Hejri et al. [43]	INbreast and private dataset	Normal, Benign, Malignant	EL, ViT	98.58	-
Alotaibi et al. [66]	BreakHis	Benign, Malign	ViT, DeiT, Soft Voting EL	98.17	-
Melekoodappattu et al. [22]	MIAS, DDSM	Malign Benign	Modifiye CNN, LBP, Gabor, LDA, UMAP, Ensemble	98.00	-
Preethi et al. [67]	MIAS	Normal, Benign, Malign	Ensemble (EfficientNet, ResNet101, VGG19)	98.30	-
Sannasi Chakravarthy et al. [26]	MIAS, INbreast	Normal, Benign, Malign	AlexNet, GoogleNet, ResNet50, DenseNet121	97.93	-
Ahmed et al. [48]	CBIS-DDSM	Malignant, Benign, Normal	VGG16, Inception V3, ResNet18, ResNet50	76.00	Grad-CAM, LIME, SHAP
Al-Tam et al. [44]	MIAS, INbreast, BUSI	Benign, Malign, BI-RADS Levels (1–6)	YOLOv8 with detect, ViT based ResNet50 with classification,	97.73	Grad-CAM
Lu et al. [68]	Special Data Set	Benign, Malign	ResNet-50	96.80	Grad-CAM
Nemade et al. [69]	DDSM, CBIS-DDSM	Malign, Benign	VGG16, InceptionV3, VGG19 / EL	98.10	-
Peta et al. [49]	BreakHis	Benign, Malignant	ESAE-Net	97.85	LIME, SHAP, Grad-CAM
**Proposed method**	AISSLabv2 [71]	Malignant, Normal	**BCECNN**	**98.75**	**Grad-CAM, LIME, LIME-Mask**

When DL models used is analyzed in the literature, it is seen that different approaches have been developed with various datasets and methodologies. However, many studies include models trained based on only a single dataset, and the evaluation of their generalization capacity is limited. Studies such as Montaha et al. [62] and Samee et al. [24] have achieved high accuracy rates using pre-trained CNN models such as VGG16, AlexNet, and GoogleNet. However, since these models do not involve the combination of multiple CNN architectures, their generalization capacity remains low. Li et al. [63] presented a more comprehensive model by combining DL and radiomics data, but the accuracy rate is relatively low (92.50%) and its clinical applicability is limited. Ahmed et al. [48] achieved only 76% accuracy despite being tested on the CBIS-DDSM dataset. This suggests that TL-based approaches alone may not be sufficient.

As shown in Table 13, significant progress has recently been made towards clinical applications through the integration of AI-based DL-oriented methodologies and XAI techniques. When evaluated in terms of the accuracy metric commonly used in breast cancer classification, the proposed BCECNN framework exhibited the highest performance compared to previous works. Moreover, not only the accuracy, but also the decision mechanisms of the model are made more transparent thanks to the combination of three different XAI techniques, which creates a significant awareness in terms of clinical applicability and expert interpretations. As a result of the expert evaluation based on the XAI visualizations, it was observed that the decision processes of the proposed method overlapped with clinically relevant regions for breast cancer diagnosis.

Because the AISSLab dataset used in this study has only recently been published, performance evaluations using this dataset are currently limited. Therefore, to assess the effectiveness of the proposed method, a comparative analysis was conducted with the most recent approaches developed for breast cancer diagnosis in the literature. Although the fact that these studies were based on different datasets limits the possibility of a direct comparison, the proposed method demonstrates a robust and competitive performance in terms of both methodological framework and achieved results. As shown in Table 13, expert evaluations using XAI techniques are often overlooked or minimally addressed in the literature. In this study, to evaluate the interpretability of the proposed method, visual XAI outputs were thoroughly examined and interpreted by an experienced expert physician. This approach validated the model outputs not only statistically but also clinically, thereby contributing a distinctive and valuable dimension to the literature for breast cancer diagnosis.

## Conclusion

This study proposed the BCECNN model, a DL-based ensemble framework that provides high classification performance for breast cancer detection. By combining multiple CNN architectures through a majority voting mechanism and integrating XAI techniques such as Grad-CAM, LIME, and LIME-Mask, the model not only increased diagnostic accuracy but also improved interpretability, a key factor for clinical application. The proposed method achieved 98.75% accuracy on the AISSLabv2 dataset after extensive performance evaluations, surpassing many existing approaches in the literature. XAI visualizations further support the model’s transparency, allowing experts to visually validate decision processes. These contributions position BCECNN as a promising AI-based decision support tool for breast cancer diagnosis, offering both methodological robustness and clinical relevance.

### Future studies

Future studies may focus on increasing both the number and diversity of images in the AISSLab dataset to enhance the generalizability and robustness of the proposed BCECNN framework. Expanding the dataset with more comprehensive samples from diverse patient populations and imaging conditions would support better model adaptation to real-world clinical settings. In addition, the adoption of more advanced and interpretable state-of-the-art models, such as ViT [70], could be considered to improve both diagnostic performance and model transparency. Exploring such architectures in combination with XAI techniques may further strengthen the clinical applicability of future diagnostic systems.

## Data Availability

The AISSLab dataset, employed in this study for breast cancer detection research, is publicly available at 10.17632/zp8yfhvndv. In addition, the AISSLab-Subdatasets, created in this study, have been made openly accessible on Kaggle at 10.34740/kaggle/dsv/12922528 and on Mendeley Data [71], providing a valuable benchmark to ensure reproducibility, enable fair comparison of AI models, and foster advancements in breast cancer detection research.
